# The clinical value of Stress Cardiac Magnetic Resonance Imaging in symptomatic patients with low to intermediate acute coronary syndrome risk

**DOI:** 10.1186/1532-429X-18-S1-O2

**Published:** 2016-01-27

**Authors:** Wei Dong, Zhanming Fan, Yi He, Jianing Pang

**Affiliations:** 1 grid.411606.40000000417615917Nuclear Medicine, Beijing Anzhen Hospital, Beijing, China; 2 grid.411606.40000000417615917Department of Radiology, Beijing Anzhen Hospital, Beijing, China; 3 grid.50956.3f0000000121529905Cedars-Sinai Medical Center, Los Angeles, CA, USA, Cedars-Sinai Medical Center, Los Angeles, CA USA

## Background

Stress cardiac magnetic resonance (CMR) imaging has become a promising non-invasive technique that is being increasingly used to evaluate acute coronary syndrome (ACS) patients. Specifically, low- and intermediate-risk ACS patients require non-invasive imaging for risk stratification, unlike high-risk ACS patients whom may receive invasive coronary angiography (ICA) directly. This study aims to assess the diagnosis performance of stress CMR for evaluating mid- and low-risk ACS patients.

## Methods

Consecutive symptomatic patients (N = 51) were prospectively enrolled from December 2013 to April 2015, with suspected ACS and with low- to intermediate-risk of disease according to the TIMI risk score. Sequential CMR, Single-Photon Emission Computed Tomography (SPECT), and invasive coronary angiography (ICA) were performed. CMR consisted of rest and adenosine triphosphate (ATP) stress perfusion, cine imaging, and late gadolinium enhancement. Semi-quantitative CMR and SPECT images were analyzed visually. We compared the diagnostic performance of stress CMR and SPECT for detecting ACS on the per-patient and per-coronary territory basis. For diagnostic performance assessment, the area under the receiver operating characteristic curve (AUC) was calculated respectively using ICA as the reference standard.

## Results

First, for detection of ACS in low-risk populations, on the per-patient level, CMR had sensitivity of 93% and specificity of 75%, and SPECT had sensitivity of 79% and specificity of 63%. The AUC of CMR was 0.879, slightly superior to that of SPECT at 0.723 (p = 0.19, n = 22). On the per-coronary territory level, CMR had sensitivity of 90% and specificity of 87%, and SPECT had sensitivity of 68% and specificity of 83%. The AUC of CMR of 0.923 was significantly superior to SPECT at 0.774 (p = 0.04, n = 66).

Second, for detection of ACS in intermediate-risk populations, on the per-patient level, CMR had sensitivity of 88% and specificity of 75%, and SPECT had sensitivity of 84% and specificity of 75%. The AUC of CMR was 0.885 and that of SPECT was 0.900 (p = 0.78, n = 29). On the per-coronary territory level, CMR had sensitivity of 73% and specificity of 78%, and SPECT had sensitivity of 73% and specificity of 76%. The AUC of CMR was 0.770, similar to SPECT of 0.785 (p = 0.75,n = 87).

## Conclusions

Our results demonstrated that stress CMR is useful and valuable in symptomatic patients with low to intermediate ACS risk. Compared with SPECT, stress CMR performed better in low risk ACS patients. Further studies are warranted to further establish stress CMR as the method of choice for evaluating intermediate- and low-risk ACS patients.

**Figure 1:**
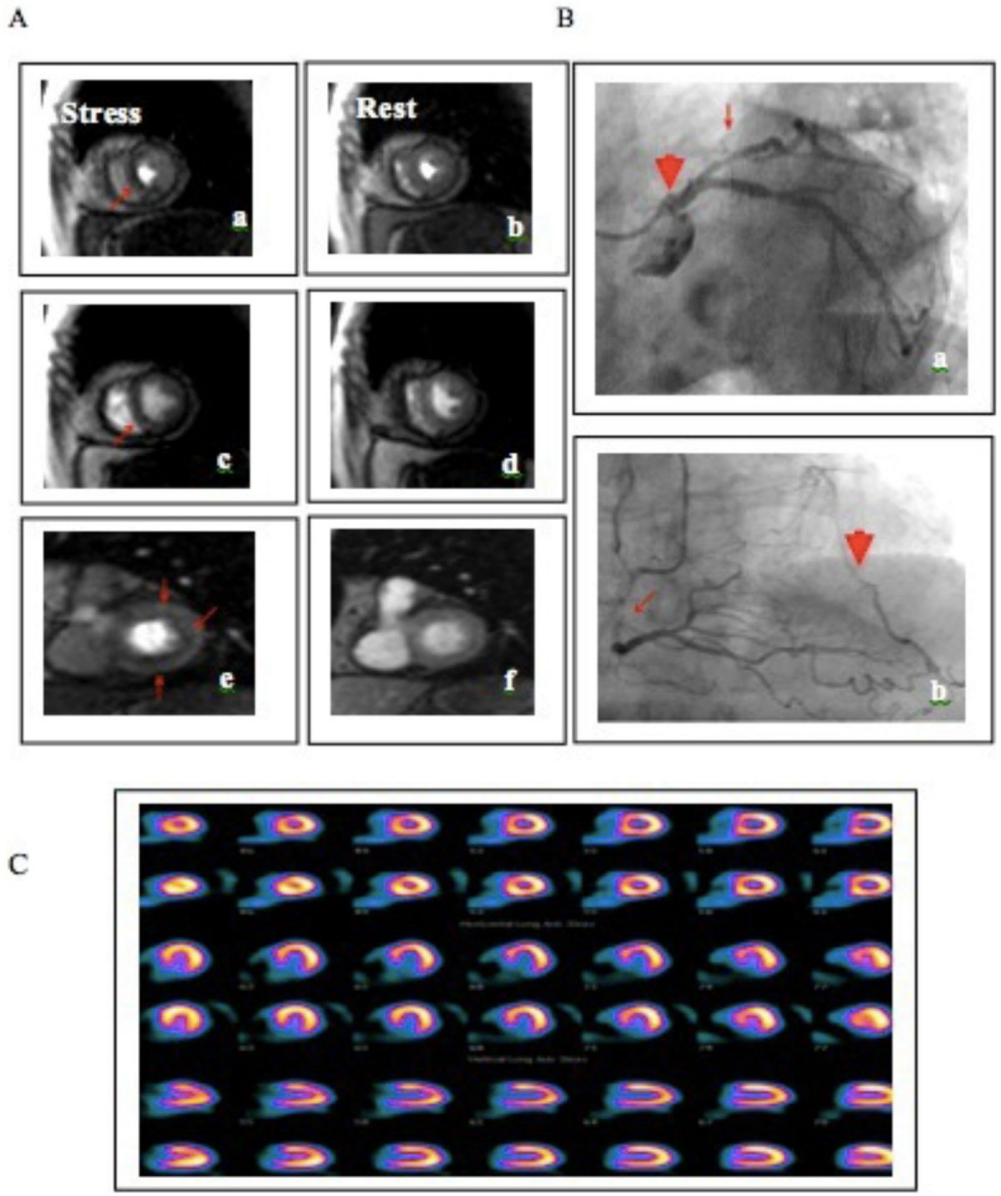
**An example of a patient complain of chest pain during stress nearly five years, aggravation a month recently**. A. Stress/rest perfusion CMR shows reversible subendocardial hypoperfusion (ischemia) at Apical septal (a), Mid inferoseptal (c), inferior wall (a, c, e), basal anterior and anterolateral wall. B CAG confirmed significant stenosis in all three coronary arteries. The occlusion of the proximal of LAB (B, a arrowhead), RCA collateral blood supply to the distal of LAD (B, b arrowhead) a severe stenosis in the LCX of 80% (B, a arrow). The middle and distal of RCA shows diffuse stenosis of 60-80% (B, b arrow). C. Stress/rest perfusion SPECT shows no significant perfusion defect. As per study protocol, SPECT in this patient was classified as a false negative, showing the potential limitations of SPECT can miss small subendocardial ischemia, and the shortcomings of detecting three balanced coronary lesions.

